# Comparison of subjectively and objectively assessed sleep problems in breast cancer patients starting neoadjuvant chemotherapy

**DOI:** 10.1007/s00520-020-05580-0

**Published:** 2020-06-19

**Authors:** Charlotte Kreutz, Jana Müller, Martina E. Schmidt, Karen Steindorf

**Affiliations:** 1 Division of Physical Activity, Prevention and Cancer, German Cancer Research Center (DKFZ) and National Center for Tumor Diseases (NCT) Heidelberg, Im Neuenheimer Feld 460, 69120 Heidelberg, Germany; 2 Faculty of Medicine Heidelberg, Im Neuenheimer Feld 672, 69120 Heidelberg, Germany; 3 Institute of Sports and Sport Science, Heidelberg University, Im Neuenheimer Feld 700, 69120 Heidelberg, Germany; 4 Working Group Exercise Oncology, Division of Medical Oncology, Heidelberg University Hospital and National Center for Tumor Diseases (NCT), Im Neuenheimer Feld 460, 69120 Heidelberg, Germany; 5 Division of Physical Activity, Prevention and Cancer (C110), German Cancer Research Center (DKFZ) and National Center for Tumor Diseases (NCT) Heidelberg, Im Neuenheimer Feld 581, 69120 Heidelberg, Germany

**Keywords:** Breast cancer, Sleep, Insomnia, PSQI, Actigraphy

## Abstract

**Purpose:**

To characterize sleep problems and to compare subjective and objective assessments in breast cancer patients starting neoadjuvant chemotherapy.

**Methods:**

Sleep characteristics of 54 breast cancer patients starting neoadjuvant chemotherapy were analyzed. Subjective sleep characteristics were assessed with the Pittsburgh Sleep Quality Index (PSQI) and objective sleep measurements with an accelerometer (ActiGraph wGT3X-BT) worn on the wrist for 7 consecutive days.

**Results:**

According to the common PSQI cut-off of 8, 10 (18.87%) of the patients were poor sleepers. ActiGraph measures did not mirror this classification as values for poor, and good sleepers did not differ significantly. Overall, Bland-Altman plots illustrated higher ActiGraph values for sleep efficiency and effective sleep time and lower values for sleep latency, compared with PSQI. For total sleep time, less disagreement between both measures was observed. Actigraphy was limited in precise identification of sleep begin and sleep latency but provided supplementary information about number and minutes of awakenings during the night.

**Conclusion:**

Subjective and objective measurement methods differed substantially in various parameters, with limitations in both methods. A combination of both methods might be most promising.

**Trial Registration:**

Clinicaltrials.gov: NCT02999074

**Electronic supplementary material:**

The online version of this article (10.1007/s00520-020-05580-0) contains supplementary material, which is available to authorized users.

## Introduction

In breast cancer survivors, sleep disturbances are among the most common long-term health issues. In general, sleep disturbances are prevalent after menopause [1], but breast cancer survivors still have significantly more insomnia than women of comparable age of the general population [2]. Among disease-free breast cancer survivors 5 years after diagnosis, 38% reported sleep problems [3]. Chemotherapy and also hot flashes, poor physical functioning, depressive symptoms, distress, quality of life, fatigue, and anxiety have been found to be significant predictors or correlates of insomnia [4]. Moreover, sleep problems are related with an increased risk of depression [5], chronic pain [6], cardiovascular diseases [7], and dementia [8].

The symptoms of insomnia comprise problems falling asleep, remaining asleep during the night, or waking up too early. Further, sleep might be too short and inadequate, light and easily disrupted, or non-restorative [9]. There are several subjective and objective ways to measure sleep disturbances. Polysomnography (PSG) is considered the gold standard for the detection of specific sleep characteristics and sleep-wake rhythm [10]. However, it is costly and not always feasible in clinical trials. Thus, actigraphy is often used for objective measurements. It offers a non-invasive method of objectively quantifying actual body movement over time in the habitual everyday situation [11]. The devices measure movements and draw conclusions about sleep based on validated algorithms [12]. On the contrary, subjective sleep characteristics are frequently assessed using questionnaires, with the Pittsburgh Sleep Quality Index (PSQI) being the most widespread one [13].

Still, there is no conclusive evidence on how well the various parameters measured by PSG, PSQI, and actigraphy agree with each other. Regarding the agreement of PSQI and PSG in healthy individuals or patients with insomnia, study findings are conflicting. One study found correlations for sleep efficiency, sleep latency, and total sleep time [14]. Two studies found only a correlation between PSQI global score and PSG sleep latency [15, 16]. Buysse et al. showed that PSQI sleep quality correlates only with PSG measures in young, but not in elderly subjects. For the young subjects, a PSQI global score > 5 resulted in a sensitivity of 98.7% and specifity of 84.4% [17]. Another study revealed no statistically significant differences in PSG measures between good and poor sleeper categorized by PSQI [18]. To the best of our knowledge, there is only one study in breast cancer survivors that compared PSG and PSQI. It did not find significant differences in standard PSG parameters (total sleep time, sleep efficiency, sleep onset and REM sleep onset latency, wake after sleep onset or sleep stage) between breast cancer survivors with none/mild or moderate/severe insomnia based on the PSQI. Only periodic limb movements were significantly correlated with subjective report of insomnia on PSQI [19].

For actigraphy, some studies have shown 91.4–96.5% minute-by-minute agreement rates with PSG regarding differentiation between sleep and wake [20–23]. In contrast, another study found an overall agreement of 53.0% [24]. A meta-analysis including 64 studies which compared actigraphy with PSG found that actigraphy overestimated total sleep time (TST) and sleep efficiency (SE) and underestimated sleep latency (SL) and wake after sleep onset (WASO) compared with PSG. These differences were larger in adults with chronic conditions compared with healthy subjects [25]. Validation studies comparing actigraphy against PSG might overestimate the validity of actigraphy due to clearer determination of time going to bed and getting up in the laboratory setting compared with measurements in the usual environment [26]. Those studies are also generally limited by the fact that a night in the sleep laboratory may not reflect usual sleep. Thus, to validate measurement of usual sleep, validation studies conducted in the normal sleep environment are needed. Most studies comparing actigraphy with PSG were in healthy subjects, with none investigating cancer patients.

Agreement between actigraphy and PSQI has been investigated in a study with 441 healthy men and women which found no correlation between actigraphy with PSQI sleep variables [27]. Further, four studies compare both measures in non-metastasized breast cancer patients [28–31], one in advanced lung cancer [32], and one in metastatic cancer patients [33], with inconclusive results: Some of these studies reported a correlation between actigraphy and PSQI with respect to TST [29, 31, 33] and SE [33] or between WASO and PSQI sleep disturbances [28, 29, 32]. Grutsch et al. also found some correlations between PSQI and actigraphy parameters, yet results differed between inpatients and outpatients [32]. On the other hand, Berger et al. observed no associations of the PSQI global score with any actigraphy measure [28]. Beck et al. divided the patients by PSQI into good and poor sleepers and found no significant difference in actigraphy variables between the two groups [30].

Overall, there are several studies that describe discrepancies between ActiGraph and PSQI, but it is currently unclear which measurement is most informative for the different sleep parameters. Further, few studies investigating subjective and objective sleep measures have been conducted in, and none only included cancer patients at the beginning of therapy when sleep might be particularly impaired by the psychologically stressful situation.

Thus, our study aimed to complement the current knowledge on subjective and objective sleep measures by (1) investigating sleep in breast cancer patients at the start of neoadjuvant chemotherapy, (2) by comparing the single sleep parameters of subjective and objective measures in detail, and (3) identifying concrete difficulties in subjective and objective measurements as well as potential sources of measurement errors especially for cancer patients at start of chemotherapy.

## Patients and methods

This study used baseline data from the BENEFIT study, a randomized controlled exercise intervention trial for breast cancer patients undergoing neoadjuvant chemotherapy (clinicaltrials.gov NCT02999074).

### Patients

Patients were eligible if they were women 18 years and older, had sufficient German language skills, had primary carcinoma of the breast, were scheduled for neoadjuvant chemotherapy (but not yet started), were willing to train at exercise facilities twice per week and to take part in the scheduled testing at the National Center for Tumor Diseases (NCT) in Heidelberg, and signed informed consent. Individuals were ineligible if they had any physical or mental conditions that would make it impossible to carry out the training program or to complete the study procedures or were already engaging in systematic intense exercise training.

## Measures

### Subjective measurement

Self-reported sleep was assessed using the PSQI questionnaire at the baseline visit, referring to the past 4 weeks. According to the scoring manual, a global score (range 0–21) and 7 subscales (range 0–3) were derived, i.e., sleep quality, sleep latency, sleep duration, habitual sleep efficiency, sleep disturbances, sleep medication, and daytime dysfunction. Higher scores indicate worse sleep. In addition, the single items provided detailed information about duration in minutes of total and effective sleep as well as self-reported latency in minutes and sleep efficiency in percent. Normative values of the scores were available from a representative sample of 4864 women from the general German population with mean age of 55.8 (± 12.2) years [34].

We used a cut-off to classify breast cancer patients into good and poor sleeper, i.e., a global score of ≤/> 8 that was previously identified to discriminate good and poor sleep in 102 breast cancer survivors [35]. Additionally, we tested the cut-off of 5, which is widely applied for the general population and previously yielded a sensitivity of 89.6% and specificity of 86.5% compared with PSG [15].

### Objective measurement

Actigraphy (ActiGraph wGT3X-BT) was used to quantify and record sleep. The device was worn for 7 consecutive days, starting at the day of the baseline visit, on the non-dominant wrist. Data were evaluated with ActiLife software Version 6.13.4. We used a sampling rate of 32 Hz, 1-minute epoch setting, and the sleep period scoring option of Cole Kripke [36]. This algorithm was specially designed for adults wearing the device on the wrist. For the sleep period detection, the algorithm of ActiGraph was used, which does not depend on wearing location [37]. The ActiGraph algorithm implemented the Tudor-Locke algorithm with an automatic sleep time period detection. ActiGraph and ActiLife provided information on the following parameters: SL (minutes), TST (i.e., time (minutes) from going to sleep until end of sleep, including latency), SE (0–100%), number and duration (minutes) of awakenings, and wake after sleep onset (WASO, number of awakening*minutes of awakening, in minutes), hereby averaging the values over the 7 days. In addition, effective sleep time (EST) was calculated as TST minus SL minus WASO.

### Complementary information

In addition to the ActiGraph assessment, the participants completed a sleep diary asking “When did you go to bed (but maybe still reading/watching TV)?”, “When did you try to fall asleep?”, and “When did you wake up?”

Further, to identify potential sources of measurement errors, we re-contacted the women with the largest differences in TST between PSQI and actigraphy by phone. We asked them about their evening routines, activity before going to bed, and changes in sleeping habits since diagnosis/therapy.

### Statistical methods

Data were analyzed using SAS Version 9.4. PSQI parameters were compared with normative mean values of the female German population using *t* tests. ActiGraph parameters were compared between good and poor sleepers categorized according to PSQI global score cut-off of 8 using *t* tests. In addition, the same analyses were performed using a cut-off of 5.

Bland and Altman limits of agreement method was used to assess agreement between quantitative parameters that were assessed by both, PSQI and ActiGraph, i.e., TST, EST, SL, and SE. Agreement between the measurements is illustrated by the differences between each pair of measurements in relation to the mean of each pair and 95% limits of agreement (mean difference ± 2 SD) [38].

## Results

### Study population characteristics

The sample characteristics are summarized in Table 1. The mean age was 49.0 (± 10.1) years, and more than half (57.5%) of the women were pre-menopausal, and 79.6% was married or partnered.

**Table 1:** Sample characteristics

Characteristics		*N* = 54
		Mean (SD)
Age		49.0 (10.1)
Body mass index		26.6 (6.7)
		N (percent)
18–< 25		40 (74.1)
25–< 30		6 (11.1)
≥ 30		8 (14.8)
Menopausal status	Pre	31 (57.5)
	Post	23 (42.6)
Married/partnered		43 (79.6)
Education		
High school or less		27 (50.0)
Some college or technical school		27 (50.0)

### Subjective sleep measurements

Table 2 shows PSQI parameters of our breast cancer study population in comparison with the normative values of the general female German population. Parameters among breast cancer patients were similar to the normative values except for sleep disturbances which were significantly higher among patients. The PSQI global score in our study population was 6.09 (± 3.10), which is higher (i.e., worse sleep) than cut-off 5 for poor sleeper. Highest mean values in subscales (0–3 scale) were for sleep quality (1.15 ± 0.66), sleep disturbance (1.13 ± 0.56), and sleep latency (1.10 ± 1.02). Widest ranges in subscales were in sleep latency and efficiency.

**Table 2:** Mean and standard deviations (SD) of sleep parameters derived from the Pittsburgh Sleep Quality Index (PSQI) questionnaire for the breast cancer study population in comparison to previously published normative values of the general German female population [30]

PSQI parameter	Study population *N* = 54	General female population *N* = 4864	P*
	Mean (SD)	Mean (SD)	
Sleep quality	1.15 (0.66)	1.22 (0.67)	0.47
Sleep latency	1.10 (1.02)	1.20 (0.97)	0.49
Sleep duration	0.62 (0.72)	0.63 (0.85)	0.88
Habitual sleep efficiency	0.87 (0.93)	0.80 (1.03)	0.61
Sleep disturbance	1.13 (0.56)	0.67 (0.59)	< 0.0001
Sleep medication	0.17 (0.61)	0.20 (0.63)	0.69
Daytime dysfunction	0.79 (0.53)	0.81 (0.67)	0.82
Global score	6.09 (3.10)	5.54 (3.58)	0.23
Sleep latency (minutes)	24.45 (25.74)		
Total sleep time (minutes)	493.37 (62.75)		
Effective sleep time (minutes)	404.77 (63.47)		
Sleep efficiency (%)	88.21 (4.65)		

*PSQI* Pittsburgh Sleep Quality Index; range of PSQI subscales is 0–3, range of PSQI global score is 0–21, lower values indicate better sleep; *t test; *H0* mean of study population = normative mean value

### Objective sleep measures

ActiGraph measurements are presented in Table 3. The mean SE of 88.21 (± 4.65%) is typically considered as normal sleep efficiency (cut-off, 85% [39]). None of the parameters differed significantly between good and poor sleepers classified according to the PSQI global score, neither using cut-off 8 (Table 3) nor cut-off 5 (Supplement 1). According to the cut-off of 5, 52.83% of patients were classified as poor sleepers, to a cut-off of 8 18.87%.

**Table 3:** Mean and standard deviation (SD) of ActiGraph parameters overall and stratified by good and poor sleepers according to the PSQI Global Score (cut-off, 8)

ActiGraph parameter	Total *N* = 53	Categorized by PSQI as:		
		Good sleepers *N* = 43	Poor sleepers *N* = 10	*p* value
	Mean (SD)	Mean (SD)	Mean (SD)	
Sleep latency (minutes)	1.13 (0.72)	1,16 (0,74)	1,01 (0,67)	0,57
Total sleep time (minutes)	490.35 (59.35)	490,41 (58,08)	490,08 (67,88)	0,99
Sleep efficiency (%)	88.21 (4.65)	88,07 (4,92)	88,84 (3,41)	0,64
Wake after sleep onset (minutes)	54.49 (16.32)	55,24 (17,01)	51,29 (13,23)	0,50
Number of awakenings	14.16 (3.90)	14,37 (3,99)	3,23 (3,50)	0,41
Minutes of awakenings	4.14 (1.09)	4,08 (1,10)	4,36 (1,05)	0,48

*PSQI* Pittsburgh Sleep Quality Index, PSQI ≤ 8, good sleeper; > 8, poor sleeper; range of PSQI subscales is 0–3; range of PSQI global score is 0–21; lower values indicate better sleep

### Agreement between subjective and objective sleep measures

**Fig. 1:**
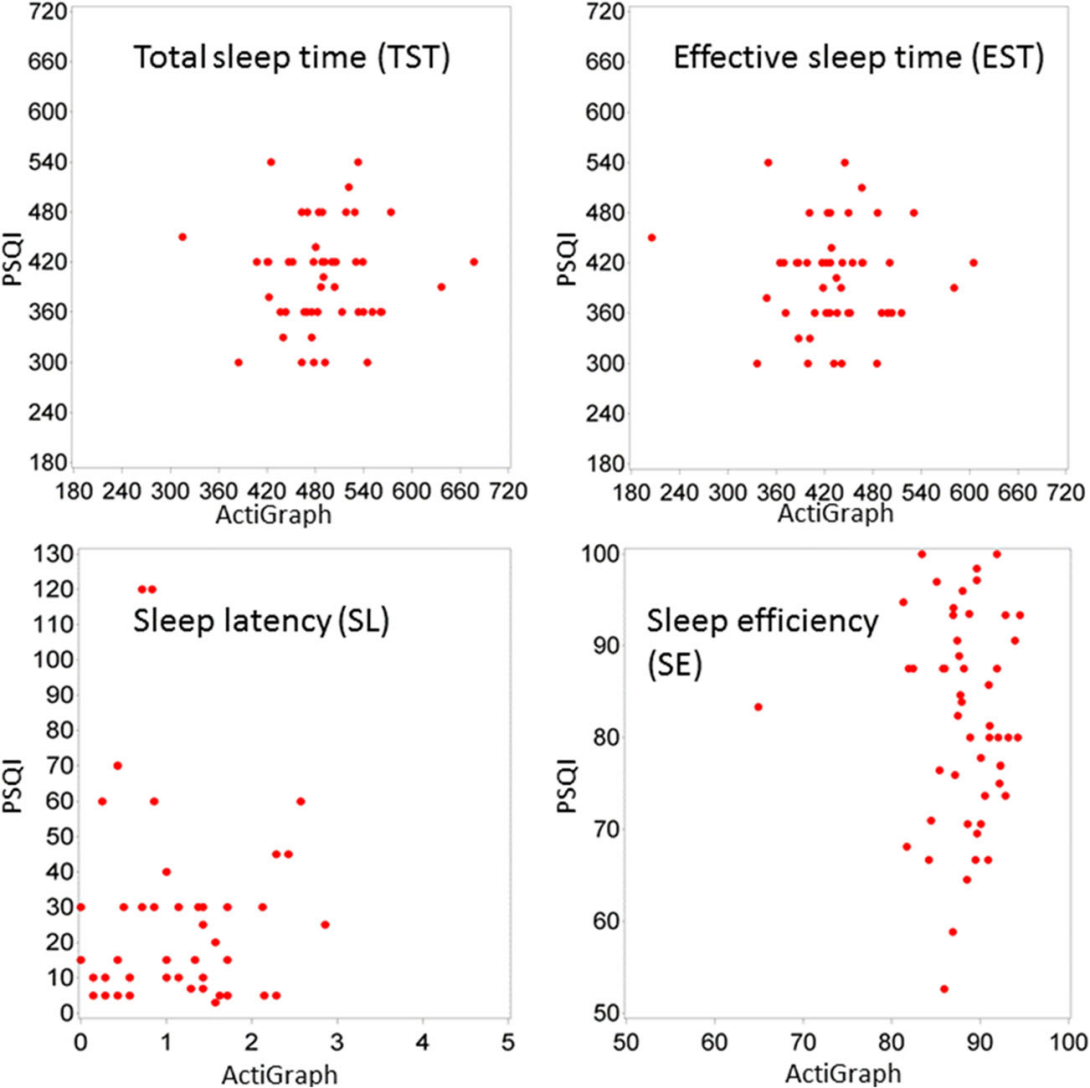
Scatter plots of PSQI and ActiGraph of total sleep time (TST) (PSQI, bedtime to get up time; actigraphy, total sleep time), effective sleep time (EST) (PSQI, effective sleep time; actigraphy, total sleep time minus WASO), sleep latency (SL), sleep efficiency (SE)

**Fig. 2:**
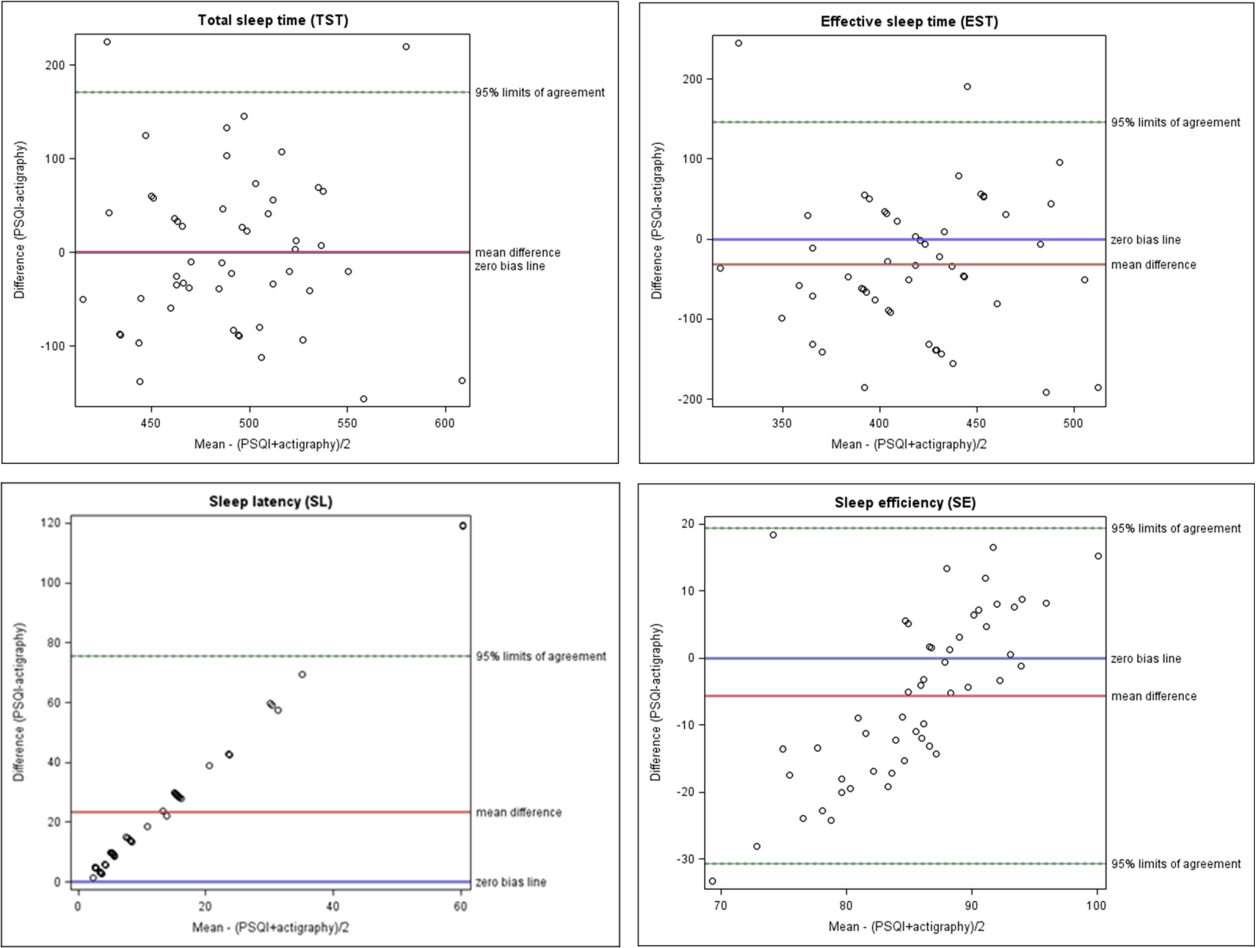
Bland-Altman plots of PSQI and actigraphy of total sleep time (TST) (PSQI, bedtime to get up time; actigraphy, total sleep time), effective sleep time (EST) (PSQI, effective sleep time; actigraphy, total sleep time–WASO), sleep latency (SL), sleep efficiency (SE)

Scatter plots of ActiGraph against PSQI values for the different sleep parameters are presented in Fig. 1. Bland-Altman plots for SE, EST, TST, and SL are shown in Fig. 2. Regarding SE, the Bland-Altman plot illustrates that on average the ActiGraph yielded higher values. Yet, for high SE, the PSQI yielded higher values than ActiGraph, whereas for low SE, it tended to be vice versa. After z-transformation, however, there was still poor agreement between both measures (Supplemental Fig. S2). Regarding SL, the Bland-Altman plot showed that there was strong disagreement between both measures with a marked underestimation of SL by ActiGraph, as also indicated by the mean of 24.45 (± 25.74) minutes of the PSQI values (Table 2) compared to 1.13 (±0.72) minutes of the ActiGraph (Table 3). In contrast, TST showed no systematic bias but substantial disagreement, i.e., 95% limits of agreement were very wide, and in 44% of participants, the PSQI and ActiGraph measures of TST differed by more than 60 min. The EST, again, was on average higher estimated by ActiGraph with wide limits of agreement.

Adjusting bedtime and wake up time in the ActiGraph data with the help of the sleep diary did not change the results substantially (data not shown).

### Qualitative assessments

In order to better understand the disagreement in TST between the two measurements, 15 women with the largest differences were re-contacted, of whom 12 reached for a phone interview. The re-contact took place between 3 and 10 months after the subjective/objective measurement. Of the 12 women, 11 reported that they routinely spent their evenings watching TV or reading and one was active before going to bed. The typical time to get to rest was around 8 pm. The women said they had not changed their evening routine and sleeping habits since diagnosis or start of therapy but described problems falling asleep and frequent awakening at night. Discrepancies in TST between PSQI and actigraphy were up to 257 min (mean 172 min). It turned out that the women with a substantially higher TST according to actigraphy compared with self-report were already resting and reading/watching TV several hours before sleeping, indicating that ActiGraph falsely counted this resting time already as sleep time.

## Discussion

This study compared self-reported and objectively measured sleep problems in breast cancer patients starting neoadjuvant chemotherapy of whom 18.87% were classified by PSQI as poor sleepers according to a cut-off value of 8. The classification was not confirmed by actigraphy. There were large differences between the two measurements. Bland-Altman analyses illustrated substantial lower estimates of SL as well as higher estimates of SE and EST by ActiGraph compared with PSQI. TST showed no clear bias.

Our study population had a mean PSQI global score of 6.09, which is slightly (yet not statistically significantly) above the mean of 5.54 among women of the general German population. Considering, in addition the younger age of the study population, the breast cancer patients might have increased sleep problems. However, the value is slightly lower than in other studies with breast cancer patients before the start of chemotherapy (6.8 [30] and 7.0 [29]). Only sleep disturbances were significantly higher compared with the general German population. However, the value we measured is slightly lower than in comparable studies (1.57 [30] and 1.4 [29]). Six of 7 subscales in our population agree with these studies. However, sleep medication was less frequent in our study (score 0.17) than in the other two studies that had been conducted in the USA (0.64 [30] and 0.95 [29]).

We measured a slightly higher ActiGraph SE of 88.21% in our breast cancer population compared with other studies with oncological patients using ActiGraph (between 78 and 85%) [30, 40–44]. This matches the PSQI result indicating that our study population had slightly less sleep problems compared with other cancer populations. However, our ActiGraph measures largely agree with other studies regarding WASO and number and minutes of awakenings [30, 40–44]. Further, the mean TST was 493 min, which is close to the results of Beck et al. with 470 min [30], but another study had measured a mean TST of only 360 min [29]. Possible causes contributing to heterogeneity in results are different wearing periods, device settings, algorithms, and adjustments. The length of the wearing periods in studies ranged from 48 h [40] to 5 weeks [41]. It is recommended that 7 days or more are assessed to measure TST, because sleep time during weekend nights is typically longer than during week nights [45]. Furthermore, scoring algorithms, devices, and modes for interpreting the data might vary [46].

Our results indicate substantial disagreement in breast cancer patients starting neoadjuvant chemotherapy between PSQI and ActiGraph measures especially regarding SL but also for SE, TST, and EST. Similar results were already shown in a study by Lemola et al. for healthy adults [27]. The parameters did not show significant agreements between the two instruments. Measurement errors in both instruments may contribute to inconsistencies but appear to be of different severity, as discussed below.

The most pronounced disagreement, concerning the measurement of SL, seems clearly due to an inability of actigraphy to appropriately assess latency. The maximal SL assessed by ActiGraph was 2.9 min, whereas it was 120 min for PSQI. Our study population wore the ActiGraph the whole day. Thus, the instrument needed to identify by its algorithms is the time point of going to sleep. If a patient was lying quietly in bed but still was awake, ActiGraph interpreted this time often falsely as sleep. On the other hand, if a patient was turning and tossing (Supplement S3) around in bed, because she could not fall asleep, ActiGraph often identified this time not as phase of intended sleep, i.e., falsely postponed the time point of going to sleep. The lack to identify SL also impairs the correct estimation of SE. Our results are in line with the findings from actigraphy comparisons with PSG, which also indicated underestimation of SL and overestimation of SE [25].

Likewise, ActiGraph appeared unable to recognize if patients woke up too early (Supplement S4) and could not fall asleep again, although they wanted and tried to do so. If they rolled around restlessly in their beds or even went to toilet in-between, the device sometimes seemed to count this no longer as sleep period, i.e., ignored the problem of waking up too early and hence overestimated SE.

Further, ActiGraph often interpreted sitting or lying quietly (e.g., reading, watching TV) (Supplement S5) before going to sleep already as sleep, hence overestimated TST and hereby again contributed to measurement error with regard to SE. On the other hand, in some cases, this led to underestimation of SE: between watching TV (which falsely was identified as sleep by ActiGraph) and going to sleep, the patient was very active, which was falsely counted as WASO (Supplement S6), i.e., suggesting a long sleepless period and hence underestimating SE. For several patients, it seemed that actigraphy did not discover that they were waking up during night but lying quietly in bed unable falling asleep again, e.g., due to worries and thoughts circling. That is, actigraphy was overestimating SE.

A possible advantage of ActiGraph is the detection of waking phases that the person may not remember the next day.

To capture the time of start and stop of intended sleep, we had asked our patients to keep a sleep diary during the period of objective measurement. However, the patients’ reporting appeared to be not always reliable. Patients confused time when they intended to sleep with time when they fell asleep or had forgotten to note the time and filled the diary retrospectively. Thus, correcting the start and stop times using the patient self-reported times might introduce other errors. In addition, for larger studies, this would be very time-consuming. To overcome the difficulties of ActiGraph in recognizing bedtime and wake up time, and thus improve measurement of TST, SL, and too early awakening, it would be helpful to have a bottom to record significant events when going to sleep and getting up. The device used in our measurement did not have this function, but there are already actigraphs where such a bottom is available.

The subjective measures also are prone to several measurement errors. The PSQI scores may be limited by recall bias, because sleep issues of the last 4 weeks are assessed. In addition, fluctuation in bedtime and get up time makes it difficult to report “typical” times and durations. This can vary greatly from weekday to weekend.

Further, patients who suffer occasionally from sleep problems may tend to record the sleep characteristics of nights with poor sleep. In some cases, it seemed that the reported EST reflected the nights when the patient was having trouble sleeping through, although the patient recorded in the question on sleep disturbances that she had such problems only twice or less per week. The same applied for SL. Overall, this may have led to an underestimation of SE. It has been observed in previous studies that many patients with insomnia overestimate SL and underestimate TST relative to PSG and actigraphy [47].

Finally, short awakings are commonly not remembered the next morning [48]. Up to about 20 of such short waking periods during the night are typical and not considered as sleep disturbance [49]. Yet, frequent short or longer awakenings may impact sleep quality. Not taking these waking periods properly into account in the estimate of the effective sleep duration will result in an overestimation of SE.

The analysis of agreement is limited by different time periods covered by subjective and objective measurements. The PSQI refers to the last 4 weeks, while the ActiGraph covered 7 nights. In our study population, the diagnosis was on average 21.5 days before completion of the questionnaires. So, the 4-week period may have included also some time before cancer diagnosis. However, if sleep pattern had changed within the last 4 weeks due to the diagnosis, it is likely that patients recorded rather the actual sleep characteristics. A further limitation is the timing of the first dose of chemotherapy. In some cases, the wearing period of the ActiGraph was completed before the first chemotherapy; in others the first dose fell within this period. However, we did not find clear differences between sleep parameters at days before and after chemotherapy application (data not shown). PSQI was completed before the beginning of the therapy.

## Conclusion

In summary, many breast cancer patients have sleep disorders at the beginning of neoadjuvant chemotherapy. Especially PSQI sleep disturbance was significantly higher compared with the general German female population. Subjective and objective measurements in our study population differed largely from each other. Both measures were prone to several measurement errors. However, to assess sleep latency, the PSQI appeared to be the better choice. Further, self-reported parameters seemed to better reflect problems such as falling asleep, or waking up too early, and thus to better capture sleep efficiency. Advantages of ActiGraph were the assessment of the number and duration of awakenings, which cannot be assessed by self-reported measures. Therefore, both methods could be used complementary in order to identify different sleep parameters.

## Electronic supplementary material

ESM 1 (DOCX 966 kb)
