# Supplementary material for: Comparison of subjectively and objectively assessed sleep problems in breast cancer patients starting neoadjuvant chemotherapy
Source: Support Care Cancer. 2020 Jun 19;29(2):1015–23. doi: 10.1007/s00520-020-05580-0 (PMC7767899; doi:10.1007/s00520-020-05580-0)
Supplement: Supplementary file 1 — (DOCX 966 kb) [file 520_2020_5580_MOESM1_ESM.docx]

**Supplement 1:**

Mean and standard deviation (SD) of ActiGraph parameters overall and stratified by good and poor sleepers according to the PSQI Global Score (cut-off 5)

| ActiGraph parameter | Total N = 53 | Categorized by PSQI as: | | |
| --- | --- | --- | --- | --- |
|  |  | **Good sleepers**  N = 25 | **Poor sleepers**  N = 28 | **p-value** |
| Sleep Latency (minutes)  Total Sleep Time (minutes)  Sleep Efficiency (%)  Wake after Sleep Onset (minutes)  Number of Awakenings  Minutes of Awakenings | *Mean (SD)*  1.13 (0.72)  490.35 (59.35)  88.21 (4.65)  54.49 (16.32)  14.16 (3.90)  4.14 (1.09) | *Mean (SD)*  1.19 (0.64)  487.66 (68.42)  87.56 (5.77)  57.08 (19.13)  14.64 (4.53)  4.19 (1.26) | *Mean (SD)*  1.07 (0.80)  492.47 (52.04)  88.80 (3.36)  52.18 (13.28)  13.73 (3.26)  4.08 (0.93) | 0.54  0.76  0.35  0.28  0.40  0.72 |

PSQI = Pittsburgh Sleep Quality Index, PSQI ≤ 5: good sleeper, >5: poor sleeper; range of PSQI subscales is 0 -3, range of PSQI global score is 0 -21, lower values indicate better sleep

**Supplemental figure S2:**

**
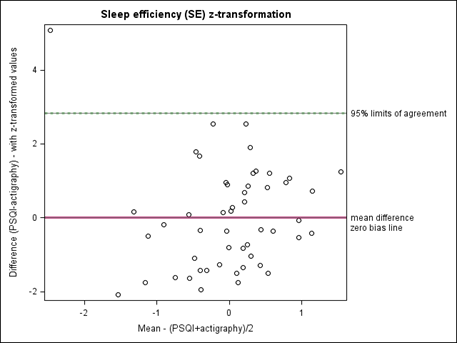
**

**Supplemental Fig. S2** Bland-Altman plots of PSQI and actigraphy of sleep efficiency (SE) z-transformed

**Supplement S3:**

If a patient was **turning and tossing** around in bed, because she could not fall asleep, ActiGraph often identified this time not as phase of intended sleep, i.e. falsely postponed the time point of going to sleep. The lack to identify sleep latency (SL) also impairs the correct estimation of sleep efficiency (SE).

**ActiGraph example:**

In bed time according ActiGraph: 22:43


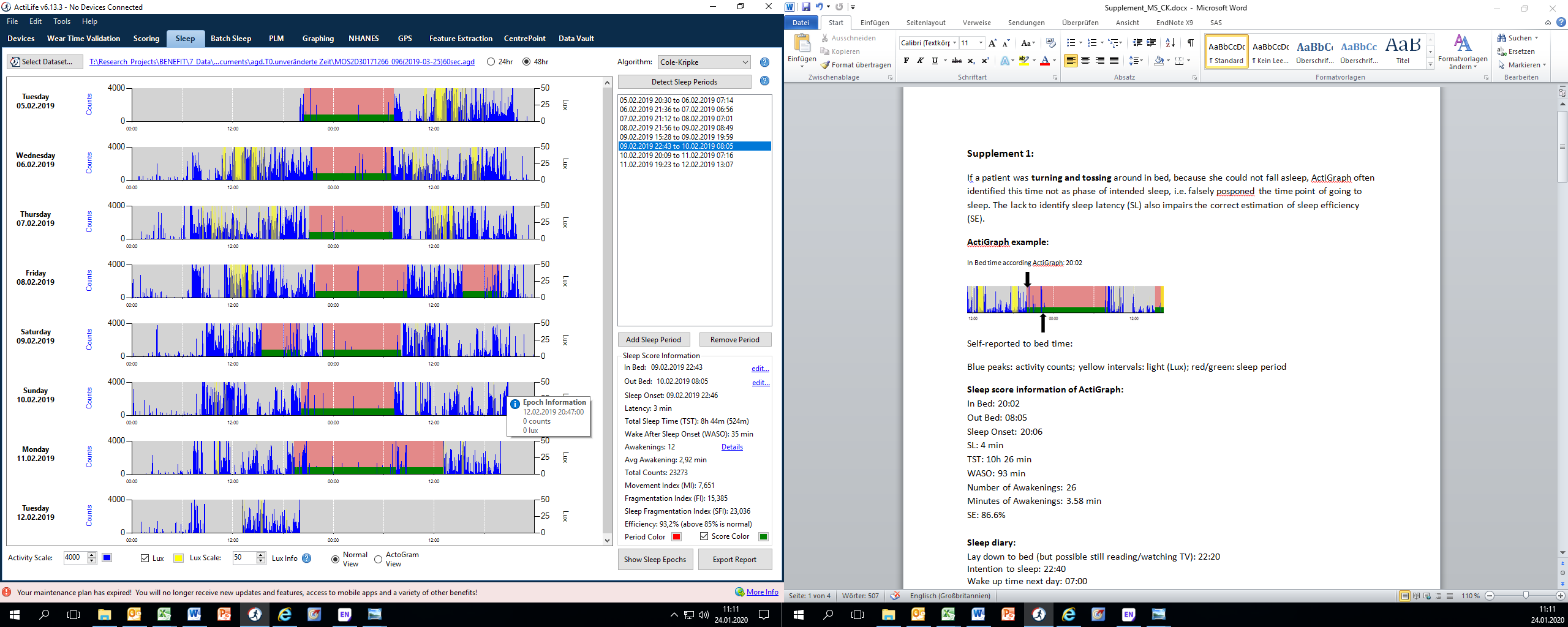


Self-reported to bed time: 22:00

Blue peaks: activity counts; red/green: sleep period

**Sleep score information of ActiGraph:**

| In Bed | 22:43 |
| --- | --- |
| Out Bed | 08:05 |
| Sleep Onset | 22:46 |
| Sleep Latency (SL) | 3 min |
| Total Sleep Time (TST) | 8 h 44 min |
| Wake after Sleep Onset (WASO) | 35 min |
| Number of Awakenings | 12 |
| Minutes of Awakening | 2.96 min |
| Sleep Efficiency (SE) | 93.2% |

**Sleep diary:**

| Lay down to bed | 21:45 |
| --- | --- |
| Intention to sleep | 22:00 |
| Wake up time next day | 05:45 |

**Supplement S4:**

Likewise, ActiGraph appeared unable to recognize if patients **woke up too early** and could not fall asleep again, although they wanted and tried to do so. If they rolled around restlessly in their beds or even went to toilet in-between, the device sometimes seemed to count this no longer as sleep period, i.e. ignored the problem of waking up too early and hence overestimated sleep efficiency (SE).

**ActiGraph example:**

Out of bed according to ActiGraph: 03:08


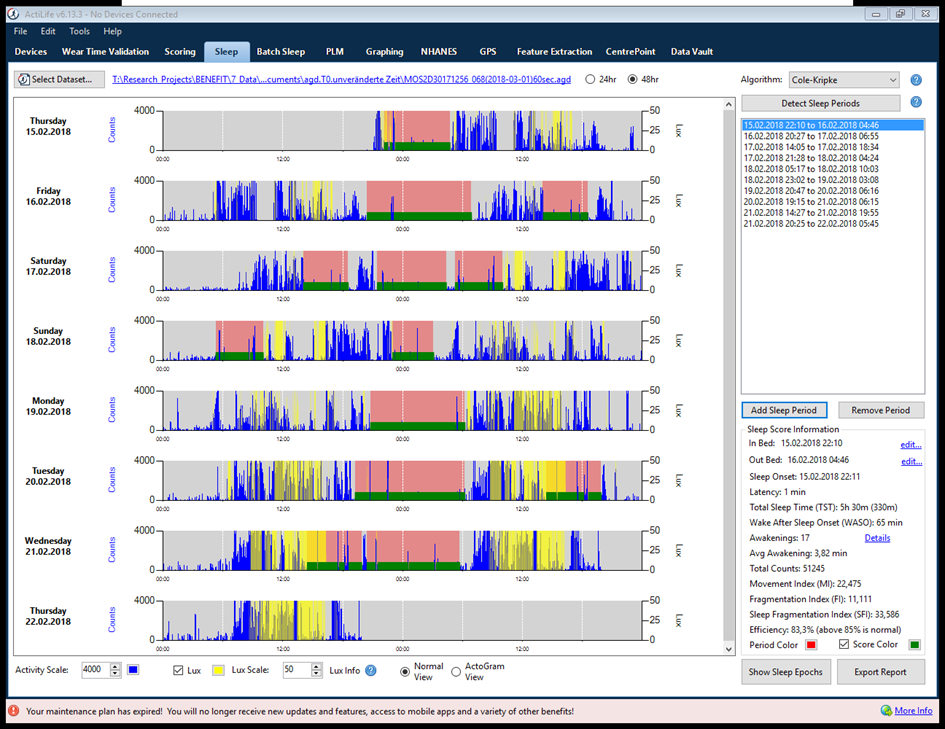


Self-reported sleep end: 04:30

**Sleep score information of ActiGraph:**

| In Bed | 23:02 |
| --- | --- |
| Out Bed | 03:08 |
| Sleep Onset | 23:02 |
| Sleep Latency (SL) | 0 min |
| Total Sleep Time (TST) | 3 h 38 min |
| Wake after Sleep Onset (WASO) | 28 min |
| Number of Awakenings | 6 |
| Minutes of Awakening | 4.67 min |
| Sleep Efficiency (SE) | 88.6% |

**Sleep diary:**

| Lay down to bed | 20:00 |
| --- | --- |
| Intention to sleep | 23:00 |
| Wake up time next day | 04:30 |

**Supplement S5:**

ActiGraph often interpreted sitting or **lying quietly (e.g. reading, watching TV**) before going to sleep already as sleep, hence overestimated total sleep time (TST) and hereby again contributed to measurement error with regard to sleep efficiency (SE).

**ActiGraph example:**

Sleep onset according to ActiGraph: 20:27


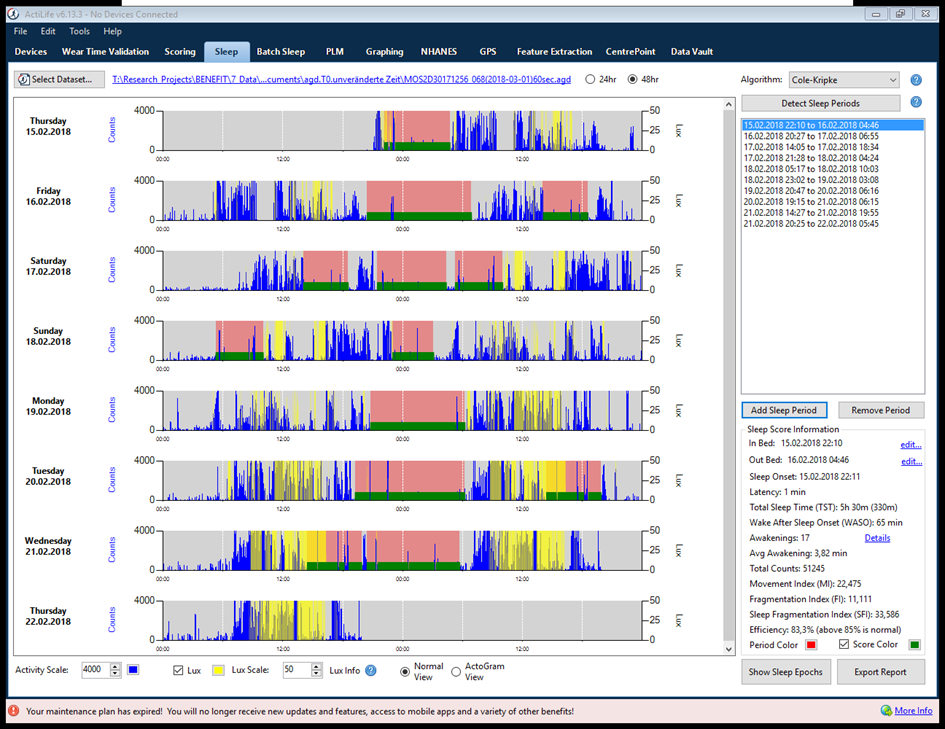


Self-reported intended sleep start: 22:00

**Sleep score information of ActiGraph:**

| In Bed | 20:27 |
| --- | --- |
| Out Bed | 06:55 |
| Sleep Onset | 20:27 |
| Sleep Latency (SL) | 0 min |
| Total Sleep Time (TST) | 10 h 04 min |
| Wake after Sleep Onset (WASO) | 24 min |
| Number of Awakenings | 2 |
| Minutes of Awakening | 12 min |
| Sleep Efficiency (SE) | 96.2% |

**Sleep diary:**

| Lay down to bed | 20:00 |
| --- | --- |
| Intention to sleep | 22:00 |
| Wake up time next day | 07:30 |

**Supplement S6:**

In some cases, between watching TV (which falsely was identified as sleep by ActiGraph) and going to sleep the patient had been very active, which **was falsely counted as wake after sleep onset (WASO)**, i.e. suggesting a long sleepless period which led to underestimation of sleep efficiency (SE).

**ActiGraph example:**

Sleep onset according to ActiGraph: 22:13


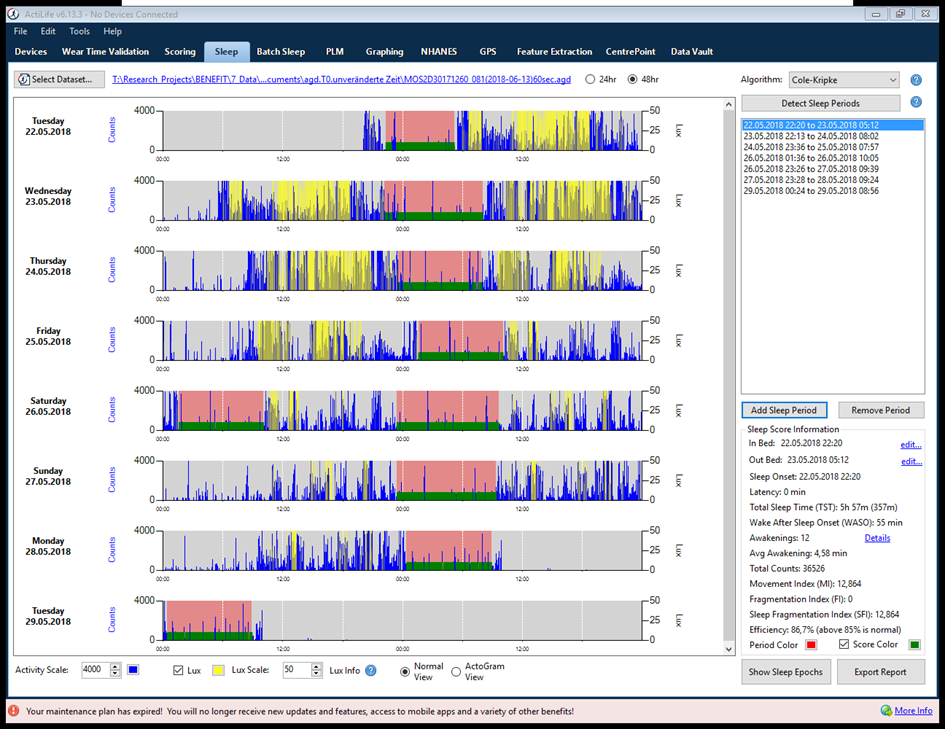


Self-reported intended sleep start: 24:00

**Sleep score information of ActiGraph:**

| In Bed | 22:13 |
| --- | --- |
| Out Bed | 08:02 |
| Sleep Onset | 22:16 |
| Sleep Latency (SL) | 3 min |
| Total Sleep Time (TST) | 8 h 11 min |
| Wake after Sleep Onset (WASO) | 95 min |
| Number of Awakenings | 22 |
| Minutes of Awakening | 4.32 min |
| Sleep Efficiency (SE) | 83.4% |

**Sleep diary:**

| Lay down to bed | 22:30 |
| --- | --- |
| Intention to sleep | 24:00 |
| Wake up time next day | 08:30 |
